# Slow to Respond: A Rapidly Progressive Case of Sporadic Creutzfeldt-Jakob Disease

**DOI:** 10.7759/cureus.53381

**Published:** 2024-02-01

**Authors:** Jasveen Kaur, Ming Tuen Lam, Sehajpreet Singh, Navjot K Somal

**Affiliations:** 1 Internal Medicine, Maimonides Medical Center, New York, USA; 2 Cardiology, Maimonides Medical Center, New York, USA

**Keywords:** rt quic, 14-3-3 protein, neurodegenerative disorders, prion diseases, creutzfeldt–jakob disease

## Abstract

Creutzfeldt-Jakob disease (CJD) is a rapidly progressive, fatal neurodegenerative disorder caused by prion proteins. In about 85% of patients, CJD occurs as a sporadic disease with no recognizable pattern of transmission. Sporadic CJD (sCJD) can present with rapid cognitive and functional decline, memory deficits, myoclonus, pyramidal and extrapyramidal signs, and visual deficits. The large spectrum of phenotypic variability has made the recognition of prion diseases difficult, and given the rare incidence, it is not uncommon for it to be missed as a potential diagnosis.

We present a highly unusual case of a 76-year-old woman with rapidly progressive sCJD who died within five weeks of presentation. Our case demonstrates a typical sequence of symptoms, with rapidly progressive dementia and cerebellar signs at disease onset and myoclonus later in the disease course.

## Introduction

Creutzfeldt-Jakob disease (CJD) belongs to a family of prion diseases or transmissible spongiform encephalopathies, which can cause several fatal neurodegenerative disorders in humans and animals. It occurs worldwide, with an annual incidence of one to two cases per million and a median age of onset of 67 years [1]. Normal cellular prion protein (PrP) is found on the membranes of cells throughout the body, even in healthy people. Central to the pathogenesis of all prion diseases is the post-translational conversion of a normal chromosome (PRNP gene)-encoded prion protein (PrPC) into an abnormal protease-resistant form (PrPSc) that can convert additional isoforms into itself and aggregate to produce neurotoxicity [2]. Creutzfeldt-Jakob disease is divided into four types: sporadic, genetic, variant, and iatrogenic. Sporadic CJD occurs in 85% of cases, and the mean survival is four to eight months. Genetic CJD occurs in 10%-15% of cases and is caused by an autosomal dominant mutation in the PRNP gene encoding prion protein. Variant CJD is caused by the consumption of infected beef, and iatrogenic CJD can be caused by contamination during brain surgery, corneal transplants, or dura mater grafts. Sporadic CJD remains the most common subtype.

## Case presentation

A 76-year-old woman with a medical history of hypertension presented with an altered mental status. Per her family, the patient had a mechanical fall two weeks ago. She was also noted as being more forgetful, confused, and repeating words. An altered mental status workup was initiated. A non-contrast computed tomography (CT) of the head revealed atrophy and mild chronic microvascular ischemia changes with calcific atherosclerosis. Urinalysis was suggestive of a urinary tract infection (UTI). The patient was discharged home with antibiotics.

After one week, the patient’s daughter called to say that her mother was having difficulty walking. The patient was called back to the hospital. Despite finishing the course of antibiotics, the patient’s altered mental status did not improve, and she started using a cane to walk. Of note, she was able to walk by herself and do activities of daily living prior to this presentation. A decision was made to repeat the CT of the head. The findings were similar to before. On examination, the patient was alert and oriented, but the neurological exam was pertinent for recall 0/3, left-sided hemi-neglect, and wide-based gait. She was able to follow three-step commands. Lab work was suggestive of borderline vitamin B12 of 357. She was started on vitamin B12 supplementation.

**Figure 1:**
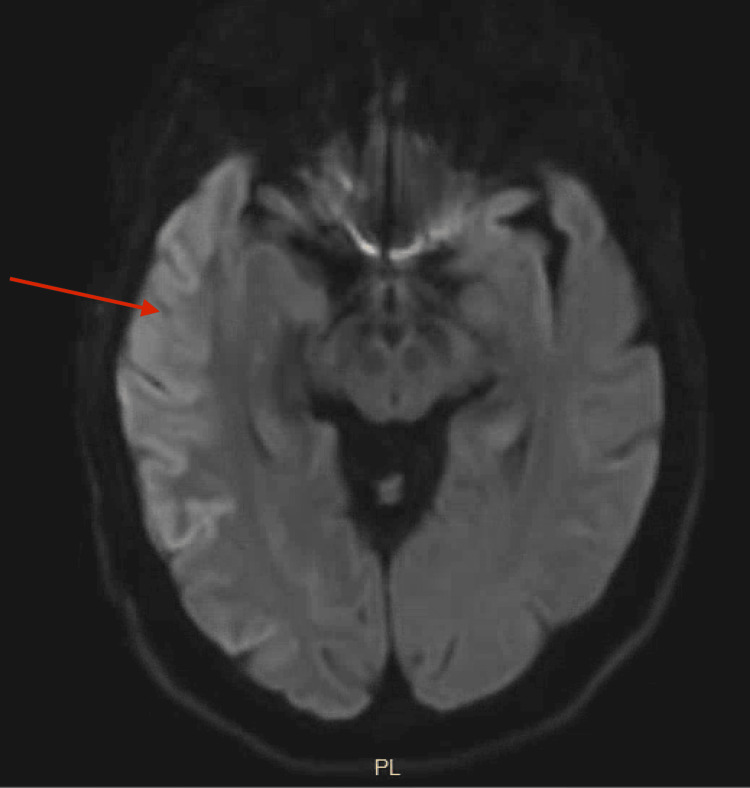
An MRI of the brain diffusion-weighted image showing hyperintensity in the right frontotemporal lobe

Electroencephalography (EEG) showed frequent right parietotemporal predominant but hemispheric sharp waves occurring in the semi-periodic pattern. A non-contrast magnetic resonance imaging (MRI) of the brain showed pyriform cortical restricted diffusion in the right temporal parietal cortex and, to a lesser extent, in the left posterior parietal temporal cortex (Figure 1).

Magnetic resonance angiography (MRA) was negative for aneurysm, vascular malformation, or significant stenosis. Differential diagnoses were thought to be focal seizures vs. encephalopathy vs. CJD. After getting the first dose of Keppra, the patient became more lethargic and unresponsive. A stroke code was called, and the patient underwent an emergent CT of the head and computed tomography angiography (CTA) of the head and neck, which were again negative. The patient was loaded with Keppra. Given the increasing unresponsiveness, a lumbar puncture was performed, and routine cerebrospinal fluid (CSF) studies were sent along with the autoimmune panel, 14-3-3, and real-time quaking-induced conversion (RT-QuIC) test. She was then placed on continuous EEG. Two days later, the patient became unresponsive with labored breathing. She was also started on pulse-dosage steroids, empirically considering alternative diagnoses such as autoimmune encephalitis. The patient was intubated for airway protection. While intubated, the patient started having myoclonic jerks. One week later, CSF results came back positive for RT-QuIC, T-tau protein >20,000 pg/ml, and 14-3-3 gamma 68,047 Au/ml, confirming the diagnosis of Creutzfeldt-Jakob disease (Table 1).

**Table 1:** The result of CSF studies (biomarkers) RT-QuIC: real-time quaking-induced conversion; CSF: cerebrospinal fluid

CSF markers	Results	Reference for non-prion disease
RT- QuIC	Positive	Negative
Tau protein	>20,000 pg/ml	0- 1149 pg/ml
14-3-3	680,47 AU/ml	<30- 1999 AU/ml

Given her poor prognosis, her family requested termination of care, made her status do-not-resuscitate/ do-not-intubate (DNR/DNI), and requested palliative extubation. But the patient died the next day, i.e., after 27 days from the date of hospitalization.

## Discussion

The diagnosis of CJD is often challenging given its variable presentation and rapid disease course. In a retrospective study of 215 CJD patients [3], dementia was observed in all patients, followed by pyramidal/extrapyramidal signs (88%), visuospatial/cerebellar signs (74%), myoclonus (62%), and akinetic mutism (46%). Dementia is also the most common initial symptom [4-6], whereas myoclonus tends to appear late in the course [7], and akinetic mutism is seen at the end stage when extensive tissue rarefaction and neuronal loss have occurred [8]. Our patient presented with rapidly progressive dementia, ataxia at onset, and myoclonus in later stages (seven days before her death, when she was already intubated).

**Figure 2:**
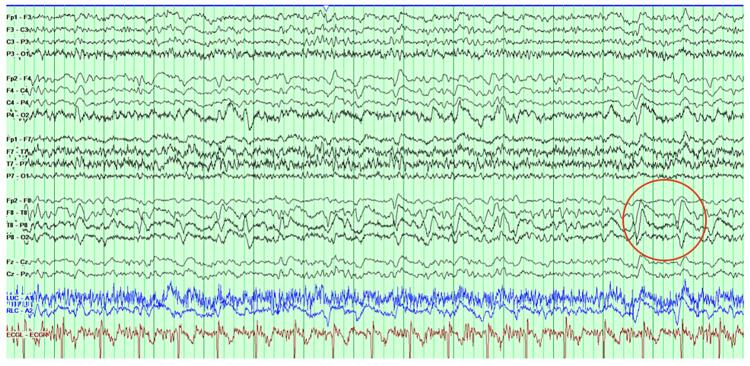
A hallmark feature of Creutzfeldt-Jakob disease is periodic sharp wave complexes (PSWCs) with triphasic morphology.

A picture of rapidly progressive dementia with a negative initial ischemic, toxic, or metabolic workup should alert clinicians to the possibility of CJD and encourage further investigations. Fluid-attenuated inversion recovery (FLAIR) and diffusion-weighted imaging (DWI) images on a brain MRI can support the diagnosis of CJD with a sensitivity of 98% and a specificity of 93% [9]. A characteristic MRI finding is gray matter hyperintensity with diffusion restriction (preferentially affecting the basal ganglia and the cortex), corresponding to the pathology of spongiform degeneration with vacuole formation in the neuropril [8, 10]. An EEG (Figure 2) is another helpful but less sensitive tool, with only 66% of patients demonstrating the typical periodic sharp wave complexes (PSWC), most of which were asymmetric and seen in the right hemisphere [11], as was the finding in our patient.

Several CSF biomarkers are also found to be of diagnostic utility in CJD. An elevated level of the stress protein 14-3-3 in the CSF has a sensitivity of 92% for CJD [12]. However, with a specificity of only 80%, one should always bear in mind the possibility of false positives as the protein can similarly be released in other disease states with rapid neuronal loss such as stroke, encephalitis, etc. The protein tau is another non-specific marker of neuronal injury with similar diagnostic performance when measured in CSF. On the contrary, RT-QuIC, a relatively new CSF assay, has produced promising results with remarkably high sensitivity (87%-91%) and specificity (98%-100%) [13]. The technique is based on the ability of the abnormal PrPSc in the CSF sample to induce the conversion of normal prion protein into itself and form aggregates that can be monitored in real-time. Given its superior performance, a positive RT-QuIC in CSF together with a compatible neuropsychiatric disorder already suffices to establish a probable diagnosis of CJD based on the CDC criteria [14], as in our current case. 

Creutzfeldt-Jakob disease remains an incurable disease, with no effective treatment available so far. Most studies quote a median survival time of around four to 13 months [4,15-19], with occasional case reports of only two months survival [20]. Our current case is unique for a rapid disease course with only 18 days to coma and 31 days to death from the onset of symptoms. Prior studies have identified several clinical, investigational, and genotypical variables that are associated with shorter survival times, including older age at onset, male sex [15, 19], the presence of myoclonus [6], the presence of visual and cerebellar signs [6], the detection of PSWCs on the EEG [6, 19], 14-3-3 positivity [6, 19], higher CSF tau levels [6], multiple myeloma (MM) homozygosity (methionine/methionine) at codon 129 of the PRNP gene, etc. On the contrary, studies did not find any correlation between MRI findings and disease duration. Multiple poor prognostic factors were identified in our patient. Of note, her CSF tau levels (ref: <1150 pg/mL) and 14-3-3 levels (ref: <2000 AU/mL) were markedly elevated at >20000 pg/mL and 68,047 AU/mL, respectively. Given that 14-3-3 and tau are markers of neuronal damage, it seems plausible that the extensive degree of neuronal damage at the time of diagnosis is the underlying factor for her ultra-short survival time.

The limitation of our case report was the absence of a brain biopsy or autopsy, as neuropathological evidence is the only way to definitively diagnose CJD. It would also have allowed further analysis of PRNP genotype, PrPSc phenotype, and disease subtype had it been performed for academic interest. However, with the advancement of the highly sensitive and specific RT-QuIC, a brain biopsy is now rarely necessary for strictly diagnostic purposes.

## Conclusions

Although there are no available treatments to cure or slow the progression of CJD, supportive treatment is the mainstay of treatment. Palliative care should be involved at an early stage to facilitate advance care planning, and family education and to help caregivers cope with the rapid decline of their loved ones. More studies need to be done to find a treatment for this fatal disease.
